# Fibroblast Growth Factor Receptors (FGFRs): Structures and Small Molecule Inhibitors

**DOI:** 10.3390/cells8060614

**Published:** 2019-06-18

**Authors:** Shuyan Dai, Zhan Zhou, Zhuchu Chen, Guangyu Xu, Yongheng Chen

**Affiliations:** 1 NHC Key Laboratory of Cancer Proteomics & Laboratory of Structural Biology, Xiangya Hospital, Central South University, Changsha 410008, Hunan, China; syandai@hotmail.com (S.D.); zhouzhan285@163.com (Z.Z.); chenzhuchu@126.com (Z.C.); 2 Key Laboratory of Chemical Biology and Traditional Chinese Medicine Research (Ministry of Education), College of Chemistry and Chemical Engineering, Hunan Normal University, Changsha 410081, Hunan, China

**Keywords:** fibroblast growth factor receptors, structure, kinase inhibitor, targeted therapy

## Abstract

Fibroblast growth factor receptors (FGFRs) are a family of receptor tyrosine kinases expressed on the cell membrane that play crucial roles in both developmental and adult cells. Dysregulation of FGFRs has been implicated in a wide variety of cancers, such as urothelial carcinoma, hepatocellular carcinoma, ovarian cancer and lung adenocarcinoma. Due to their functional importance, FGFRs have been considered as promising drug targets for the therapy of various cancers. Multiple small molecule inhibitors targeting this family of kinases have been developed, and some of them are in clinical trials. Furthermore, the pan-FGFR inhibitor erdafitinib (JNJ-42756493) has recently been approved by the U.S. Food and Drug Administration (FDA) for the treatment of metastatic or unresectable urothelial carcinoma (mUC). This review summarizes the structure of FGFR, especially its kinase domain, and the development of small molecule FGFR inhibitors.

## 1. Introduction

The human fibroblast growth factor receptor (FGFR) family consists of four members: FGFR1 to FGFR4. Despite being encoded by separate genes, the four members share high homology, with their sequence identity varying from 56% to 71% [1]. Similar to other receptor tyrosine kinases (RTKs), FGFRs are expressed on the cell membrane and can be stimulated and activated by extracellular signals. The native ligand of FGFRs is fibroblast growth factors (FGFs) [2,3,4]. The binding of FGFs drives the dimerization of FGFRs; subsequently, a transautophosphorylation event of the intracellular kinase domain is induced, followed by the activation of downstream transduction pathways [5,6]. Through triggering downstream signaling pathways, FGFRs participate in various vital physiological processes, such as proliferation, differentiation, cell migration and survival [7,8,9].

Aberrant expression of FGFRs has been shown in various kinds of solid tumors, and moreover, the aberrancy is considered an oncogenic signaling pathway [10,11,12]. It is believed that small molecules that competitively bind to the adenosine triphosphate (ATP) pocket of aberrant FGFRs while exhibiting little or no toxicity provide limitless prospects for the treatment of relevant tumors. The structure of FGFRs, especially the kinase domain, and the design of small molecular inhibitors have attracted intensive study in the past two decades. Multiple small molecule inhibitors have been developed, and some of them are currently being used in clinical trials, such as FGF401, which targets FGFR4 for the treatment of hepatocarcinoma (HCC) [13]; AZD4547, which targets FGFR1-3 for the treatment of a variety of tumors [14]. Moreover, erdafitinib (JNJ-42756493) [15] has been approved recently by U.S. Food and Drug Administration (FDA) for the treatment of mUC. More than 20 FGFR kinase/inhibitor complex structures have been determined to-date, and these structures have yielded extensive insights into the understanding of inactivation of FGFRs for related disease therapy.

## 2. Organization of FGFR

**Figure 1:**
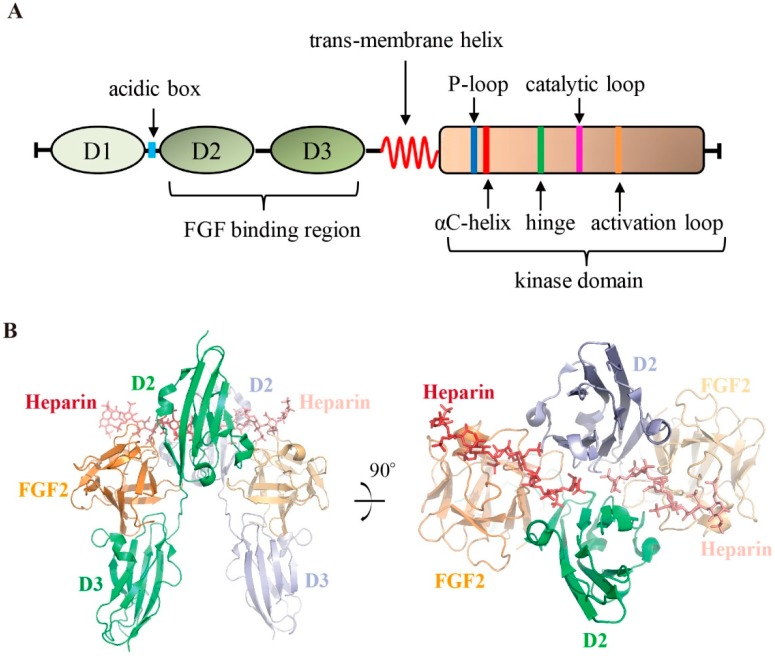
Schematic diagram of FGFRs and the structure of the FGFR extracellular domain. (**A**) Organization of FGFRs. Important functional elements are highlighted. (**B**) Crystal structure of the FGF2:FGFR1:heparin ternary complex (PDB ID 1FQ9). The two copies of FGFR1 molecules are colored in green and light blue respectively. Heparin molecules are shown in red stick representation; FGF2 (colored in orange) and FGFR1 are shown in cartoon representation.

FGFRs share a canonical RTK architecture. From the N- to the C-terminus, all four FGFR members contain a large extracellular ligand-binding domain that comprises three immunoglobulin (Ig)-like subunits (D1, D2 and D3) followed by a single transmembrane helix and an intracellular tyrosine kinase domain [1,16] (Figure 1A). The linker region between D1 and D2 contains a highly conserved motif that is rich in aspartate acids, called the acid box [17]. The detailed function of those structural units will be further introduced below.

FGFs are the native ligand for this family of kinases. Through its extracellular domain, FGFR recognizes and is stimulated by specific FGFs. The FGF binding pocket is formed by the D2 and D3 subregions [18]. There have been contradicting views regarding the stoichiometry of the FGF/FGFR complex. Schlessinger, J. et al. solved the ternary complex structure of FGF2/FGFR1/heparin [19]. With the help of heparin, FGFR1 was dimerized after the binding of FGF2 to form the complex at a symmetric 2:2:2 stoichiometry ratio; both the FGF2 and heparin molecules simultaneously contacted the two FGFR1 monomers (Figure 1B). In the FGF1/FGFR2/heparin crystal structure solved by Pellegrini et al., the complex was assembled by asymmetric 2:2:1 stoichiometry [20]. By utilizing nuclear magnetic resonance, Saxena et al. studied the interactions of FGF1(FGF2)/FGFR4/HM (HM: heparin mimetics) complex, and their results supported the formation of the symmetric mode of FGF/FGFR dimerization in solution [21]. Interestingly, although all FGFs have a heparin sulfate binding site on their surface [22,23], endocrine FGFs such as FGF21 and FGF23 show a lower binding affinity to heparin sulfate [23] and require Klotho coreceptors instead to act as cofactors for FGFR activation [24,25,26].

In addition to acting as the ligand sensor, the extracellular domain also undertakes an autoinhibitory role, which relies on regulation by D1 and the acid box [27,28]. Several studies have proposed that the acid box could competitively bind to the heparin binding site of D2 to suppress heparin binding, while D1 forms intramolecular contacts with D2-D3, thus blocking the binding of FGFs [28,29,30]. Nevertheless, the mechanisms of autoinhibition need to be further clarified.

## 3. Structure of FGFR Kinase Domain

**Figure 2:**
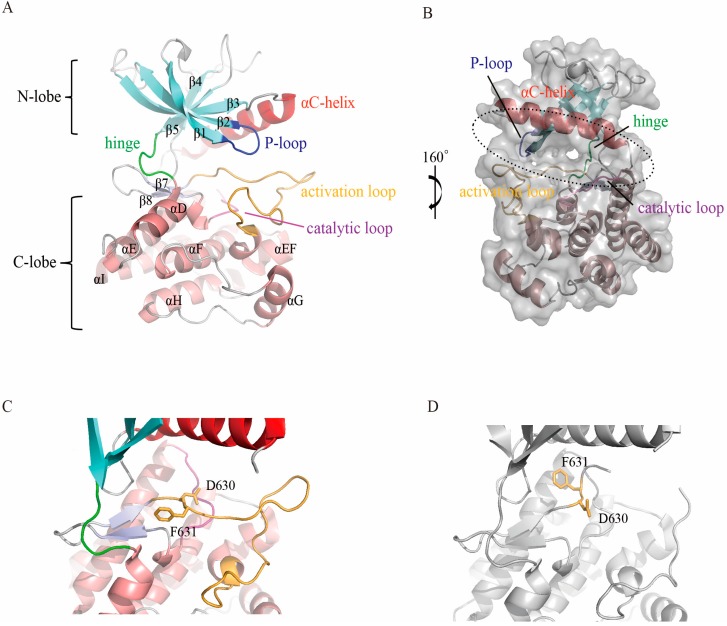
Structure of the FGFR kinase domain. (**A**) Overall crystal structure of FGFR4 in cartoon representation. The five β-sheets of the N-lobe are labeled in cyan, and the helixes of the C-lobe are colored in salmon. The αC helix (red), P-loop (blue), catalytic loop (magenta), activation loop (bright orange) and hinge (green) are highlighted. (**B**) Surface presentation of FGFR4. The ATP binding pocket located between the N- and C-lobe is indicated by the dashed circle. (**C**) DFG-out conformation of the FGFR4 activation loop. The side chains of D630 and F631 are shown in stick representation. (**D**) DFG-in status of the FGFR4 activation loop. (**A**–**C**) were prepared from PDB ID 4UXQ; (**D**) was prepared from PDB ID 5JKG.

The intracellular tyrosine kinase domain is the most well studied region of the FGFR protein. This domain exhibits the canonical bilobed architecture of protein kinases [31,32,33,34,35]. The fold of the N-terminal small lobe (N-lobe, ~100 amino acid residues) consists of a five-stranded antiparallel β-sheet (β1–β5) and the αC-helix, an important regulatory element. The C-terminal large lobe (C-lobe, ~200 amino acid residues) predominately comprises seven a helices (αD, αE, αEF, αF-αI) (Figure 2A). The active site, which is responsible for ATP and substrate protein binding, is located in a clef between the two lobes (Figure 2B).

The C-lobe folds tightly with the αF-helix to form a hydrophobic core, around which the other secondary segments are packed. In addition to the primary helixes, the C-lobe contains a short helix located between the activation loop (A-loop) and the αF-helix named the αEF-helix, which is conserved among all FGFR members as well as other protein kinases [36]. Two short β-strands (β7 and β8) (Figure 2A) between the catalytic loop and activation loop (introduced below) interact with each other and are believed to participate in the regulation of FGFR activation [37]. In contrast to the C-lobe, the N-lobe folds in a more flexible manner, which benefits the binding and release of ATP/ADP and substrates.

There are several functionally important loops in both lobes. The loop between β8 and the αEF-helix is an activation loop (A-loop), which is essential for kinase activation [38,39,40]. The conformation of the highly conserved Asp-Phe-Gly motif (DFG-motif) in the A-loop is an indicator of kinase activity status [39,41]. Generally, the DFG-motif exists in two states: the active DFG-in and inactive DFG-out conformations [42,43] (Figure 2C,D). In the DFG-in state, the aspartate residue of the DFG-motif plays an essential role in ATP binding through the coordination of all three phosphate groups of ATP, either directly or via magnesium ions, while these interactions are sterically impossible when the motif is flipped into the DFG-out conformation. Phosphorylation is catalyzed by the conserved aspartate of the His-Arg-Asp (HRD) motif in the catalytic (αE–β7) loop [44]. The glycine rich P-loop (also called the nucleotide binding loop), located between the β1- and β2-strands, folds over to enclose ATP for phosphotransfer [45]. The so-called molecular brake located at the hinge region that connects the N- and C-lobes plays a critical role in the regulation of autoinhibition and activation [46].

The catalytic activity of the kinase domain is precisely controlled. There are two general conformations for all protein kinases, including those of the FGFR family. Activation typically involves changes in the orientation of the αC-helix in the small lobe and the activation loop in the C-lobe. During the catalytic cycle, the active kinase toggles between open and closed conformations. In the open form, the kinase binds MgATP and the protein substrate, while during catalysis, the kinase adopts the closed form. Once catalysis is completed, the MgADP and phosphorylated substrate are released, and the enzyme recovers to the open conformation, preparing for the next catalytic cycle [16,47].

## 4. Characteristics of FGFR/Inhibitor Interaction

**Figure 3:**
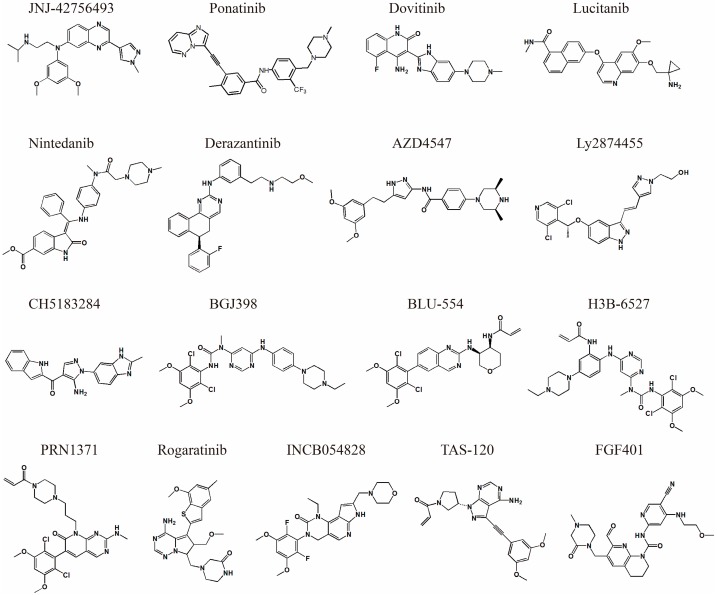
Chemical structure of FGFR small molecule inhibitors.

As noted above, aberrantly expressed FGFRs have been implicated in various tumors. Therefore, extensive work has been performed on the development of FGFR inhibitors. The inhibitors that are in clinical trials or approved by the FDA for clinical use are summarized in Table 1, and the chemical structures of those inhibitors are shown in Figure 3.

**Figure 4:**
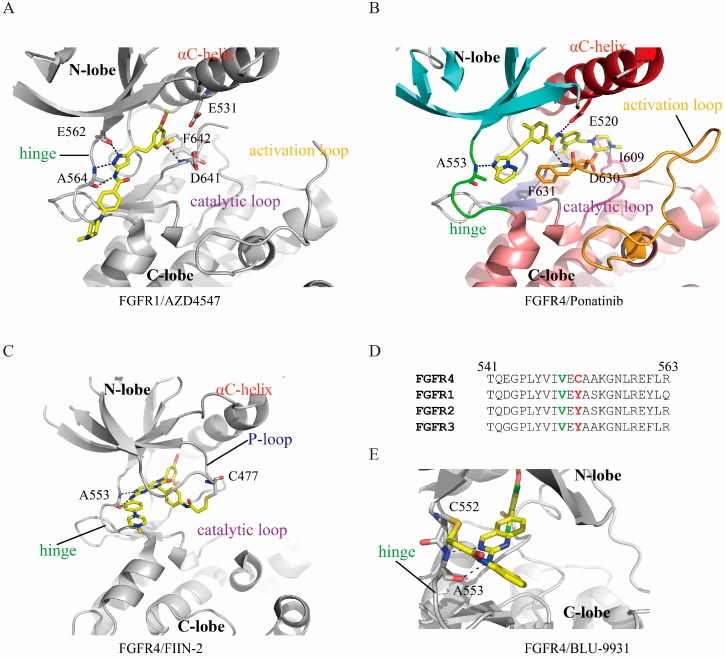
FGFRs/inhibitor interaction features. All inhibitors are presented in yellow stick representation. (**A**) Structure of AZD4547 bound to FGFR1. The side chains of A564, E562 and D641, which directly form hydrogen bonds with the inhibitor and F642 of the DFG-motif, are shown. The hydrogen bonds are indicated by dashed lines. AZD4547 binds FGFR1 into DFG-in status, the side chain of F642 points out from the ATP pocket. (**B**) The structure of FGFR4 in a complex with ponatinib. The DFG-motif of FGFR4 flipped to an out conformation with F631 benzene ring flipped into the ATP-pocket and D630 point out from the pocket. (**C**) Covalent interaction of FIIN-2 and FGFR4. The side chains of A553 and C477, which interact with ponatinib, are shown in stick representation. Covalent bond formed between C477 and the acrylamide group of FIIN-2. (**D**) Sequence alignment of the FGFR hinge region. The C552 in FGR4 is replaced by a tyrosine in the other 3 members. The gatekeeper residues which locate at the kinases hinge region and play an essential role in determining pocket accessibility for inhibitors are highlighted in green. (**E**) Structure of BLU-9931 in complex with FGFR4. Unlike the pan-FGFR covalent inhibitors, BLU-9931 targets the unique C552 of FGFR4 to form covalent interactions.

FGFR inhibitors can generally be divided into two groups according to their binding behaviors, namely, type I and type II inhibitors [58,59]. Type I inhibitors bind FGFRs in the DFG-in enzymatic active conformation in an ATP-competitive manner, while the binding of type II requires the DFG-motif to be flipped to the DFG-out state [60,61]. The X-ray crystallographic structures of AZD4547 (PDB ID 4V05) [34] and PD173074 (PDB ID 2FGI) [62] bound to FGFR1 demonstrate that these two inhibitors are type I inhibitors. Taking the FGFR1/AZD4547 structure as an example, the AZD4547 occupying the ATP pocket of FGFR1 forms a hydrogen bond with the backbone nitrogen atom of the DFG aspartate (Asp641) and forms three hydrogen bonds with the hinge residues [34] (Figure 4A). In contrast, both the DFG-motifs of FGFR4 and FGFR1 are flipped out into an inactive conformation in the FGFR4/ponatinib (PDB ID 4UXQ) and FGFR1/ponatinib (PDB ID 4V04) structures [34]. In addition to the basal interactions, a hydrogen bond formed between ponatinib and the side chain of the strictly conserved glutamate from the αC-helix (Glu520 in FGFR4 and Glu531 in FGFR1) was also observed, which is characteristic of a type II inhibitor [61] (Figure 4B). Thus, these structures reveal ponatinib to be a type II inhibitor for FGFRs. As a consequence, the flip of the phenylalanine side chain breaks the regulatory spine and creates an additional induced-fit hydrophobic pocket that allows deeper binding of the inhibitor and provides better selectivity [34,43] as well as slower dissociation kinetics [34,63].

The interaction between a small molecule inhibitor and protein kinase can be covalent (irreversible) or noncovalent (reversible) [64,65]. Typically, covalent inhibitors have a functional group known as the warhead, which can improve binding affinity and selectivity through covalent interaction with a certain residue of the target kinase [65,66]. Moreover, a well-designed warhead could provide better performance against drug resistance than reversible inhibitors [67,68]. The reported covalent reactive residues in protein kinases include cysteine [69], aspartic acid [70], lysine [71] and others [72]. For FGFRs, the conserved cysteine of the P-loop (C488 in FGFR1, C491 in FGFR2, C482 in FGFR3 and C477 in FGFR4) and the unique C552 in FGFR4 from the hinge region are the covalent binding sites. The FGFR4/FIIN-2 complex structure (PDB accession number 4QQC) is the first solved irreversible structure, where FIIN-2 formed a covalent bond through its reactive acrylamide group with the hydrosulfonyl side chain of FGFR4 C488 [73] (Figure 4C).

## 5. Current Status of Small Molecule FGFR Inhibitor Development

Designing specific small molecule inhibitors targeting protein kinases is challenging because the ATP binding pockets of the human kinome are similar [74,75]. Inhibitor research for FGFRs has gone through several stages. Initially, nonselective multiple-kinase inhibitors were developed to treat FGFR aberrations. Those nonselective inhibitors, including ponatinib [76], dovitinib [77] lucitanib [78] and nintedanib [79] (see Table 1 for details), were originally designed for other kinases and then proved to have potent inhibition activity toward FGFRs. For instance, the type II inhibitor ponatinib was originally developed to overcome the BCR-ABL T315I gatekeeper mutant and showed single-digit nanomolar binding strength to FGFR1-4 in later researches [76]. Although nonselective inhibitors might be clinically beneficial and achieve therapeutic success to some extent, the development of those inhibitors has been restricted due to the undesirable off-target toxicities [80,81].

To overcome the off-target effects of nonselective inhibitors, efforts have been made to develop FGFR-selective inhibitors (pan-FGFR inhibitors). In the earlier stage, multiple noncovalent pan-FGFR inhibitors were developed, including the well-known AZD4547 [14] and LY2874455 [48] (see Table 1 for details). Those pan-FGFR inhibitors are typically type I inhibitors. For example, AZD4547 is capable of potently inhibiting FGFR1-3 but shows negligible binding affinity to FGFR4. AZD4547 is currently in phase II clinical trials. However, preclinical data show that AZD4547 is not able to overcome the gatekeeper mutation V555M in FGFR3 [82]. Unlike AZD4547, the inhibitor LY2874455 shows inhibition efficacy against all 4 FGFRs, and the crystal structure as well as in vitro and vivo experiments confirmed that this inhibitor maintained equal inhibitory ability against the gatekeeper mutant V550M/V550L of FGFR4 [32].

Given that covalent inhibitors confer better binding kinetics and selectivity than noncovalent ones, developing irreversible inhibitors of FGFRs has attracted intensive pharmaceutical and academic attention in recent years. Since the first covalent inhibitor FIIN-1 [83], this field has achieved much progress. A number of FGFR covalent inhibitors have been developed, and some of those agents are already in clinical trials (see Table 1 for details). Moreover, several irreversible inhibitor/FGFR structures have been revealed by crystal structures, including FGFR4/BLU9931 (PDB ID: 4XCU) [84], FGFR4/FIIN-3 (PDB ID: 4R6V) [73], FGFR4/FIIN-2 (PDB ID: 4QQ5) [85], FGFR4/CGA159527 (PDB ID: 5NUD) [86], FGFR1(Y563C)/H3B-6527 (PDB ID: 5VND) [86], and FGFR1/TAS-120 (PDB ID: 6MZW) [54].

The kinase domains of FGFRs are highly homologous, with sequence identity varying from 74% to 77% [34]. Unexpectedly, most of the reported pan-FGFR reversible inhibitors tend to bind FGFR1-3 but exhibit greatly reduced potency toward FGFR4 [87]. The underlying mechanism is not quite clear. Tucker et al. proposed that the innate flexibility of the FGFR4 kinase domain might be responsible for the decrease in binding ability [34]. This feature, together with the unique C552 of FGFR4, which replaces a tyrosine in FGFR1-3, confers the opportunity to develop FGFR4-selective inhibitors [88,89] (Figure 4D). Indeed, H3B-6527 [56], BLU-9931 [84], BLU-554 [84] and FGF401 [90] were developed as FGFR4-selective covalent inhibitors that target C552 for irreversible binding (Figure 4E). Among these four molecules, FGF401 is the most interesting because the covalent bond it forms is reversible, which reduces the off-target effect and prolongs the residence time [91,92]. The crystal structure of FGF401/FGFR4 was recently reported by our laboratory (PDB ID 6JPJ) [57], and its potential utility is currently under intensive research.

In addition to the kinase domain, the ectodomains of FGFRs have also attracted intensive interests for drug discovery. Unlike the highly conserved kinase domain, the ectodomains of FGFRs are less conserved; targeting this domain may offer better isoform selectivity. The dominant strategy to target FGFR ectodomains is using monoclonal antibody/antibody-drug conjugate [93]. Several anti-FGFR monoclonal antibodies have been developed, and some of them are in clinical trials [94,95,96,97,98]. In addition, efforts have been made in the development of small molecule inhibitor targeting FGFR ectodomains. An inhibitor, SSR128129E, which allosterically binds to the ectodomain of FGFR in a non-FGF competitive manner, has been reported to inhibit FGF-induced signaling [99,100].

## 6. FGFR Gatekeeper Mutation and Drug Resistance

The long-term efficacy of kinase inhibitors in cancer treatment is often disturbed by acquired resistance. One common mechanism of resistance is generated by mutating the so-called gatekeeper residue of the kinase domain [101]. The gatekeeper mutation has been reported in various kinases, such as Bcr-Abl (T315I) [102], EGFR (T790M) [68], PDGFR (T674I) [103], FGFR1 (V561M) [104] and FGFR2 (V565I) [105]. The gatekeeper residue lies at the beginning of the hinge region and controls the accessibility of the hydrophobic pocket. Most protein kinases harbor a threonine that plays a major role in the interaction with the inhibitor by forming a critical hydrogen bond via its side chain hydroxyl oxygen. The mutation of this residue to a bulky hydrophobic amino acid, either Met or Ile, breaks the hydrogen interaction and introduces steric hindrance for inhibitor binding [76,106].

The drug resistance of FGFR gatekeeper mutations has been extensively verified, both in vitro and in vivo. For instance, the V564M mutation of FGFR2 confers the ability to resist dovitinib and BGJ398 [107]. Furthermore, an array of FGFR gatekeeper mutations have been identified in clinical samples. For example, the FGFR4 V550M mutation was detected in 13% of neuroendocrine breast carcinomas [108]. In FGFRs, the gatekeeper residue is a valine (Figure 4D); as a consequence, its side chain cannot form hydrogen interactions with inhibitors (see above context), and the resistance thus arises mainly through the introduction of steric hindrance. Several FGFR inhibitors have been shown to have the ability to overcome FGFR gatekeeper mutations. For example, Ly2874455 has almost equal binding affinity to wild-type FGFR4, FGFR4 (V550M), FGFR4 (V550L) FGFR1 (V561M), FGFR2 (V564F) and FGFR3 (V555M) [32]; FGF401 has similar affinity to wild-type FGFR4, FGFR4 (V550M) and FGFR4 (V550L) [57]; FIIN-2 shows a binding affinity loss of ~10-fold for FGFR4 (V550L) compared with the wild-type kinase [85].

## 7. Conclusions

Increasing evidence indicates that aberrant Fibroblast growth factor receptors (FGFR)signaling plays a crucial role in tumorigenesis and progression. Now, small molecule inhibitors targeting FGFRs offer a novel and effective strategy for the therapy of cancers caused by FGFR aberrations. Efforts and progress have been made in the field of FGFR inhibitor development. Some small molecules show promising antitumor activity and are evaluated in clinical trials. Recently, the pan-FGFR inhibitor erdafitinib has been approved by the Food and Drug Administration for the treatment of mUC, making it the first approved FGFR-targeted drug. However, there are still challenges in the field of FGFR inhibitor development, such as the need for more potent and selective FGFR inhibitors, and inhibitors with the ability to overcome gatekeeper mutations. Most FGFR inhibitors currently under evaluation are typical type I inhibitors that occupy only the ATP binding pocket. Development of type II FGFR inhibitors, which could be inserted deeper into the pocket, could confer better potency and selectivity. In addition, the development of covalent irreversible or covalent reversible FGFR inhibitors might be another strategy to improve safety and efficacy for cancer treatment.

## Figures and Tables

**Table 1:** FGFR inhibitors that are in clinical trials or approved by the FDA.

Inhibitor Name	Binding Features	IC50 (nM)	PDB ID	Clinical Trial Phase/Number	Reference
JNJ-42756493 (Erdafitinib)	Pan-FGFR Reversible Type I	FGFR1: 1.2 FGFR2: 2.5 FGFR3: 3.0 FGFR4: 5.7	n/a	FDA approved	[15]
AZD4547	Pan-FGFR Reversible Type I	FGFR1: 0.2 FGFR2: 2.5 FGFR3: 1.8 FGFR4: 165	4V05	Phase I/II NCT02824133	[14,34]
Ly2874455	Pan-FGFR Reversible Type I	FGFR1: 2.8 FGFR2: 2.6 FGFR3: 6.4 FGFR4: 6	5JKG	Phase I NCT01212107	[33,48]
CH5183284	Pan-FGFR Reversible Type I	FGFR1: 9.3 FGFR2: 7.6 FGFR3: 22 FGFR4: 290	5N7V	Phase II/III NCT03344536	[49]
NVP-BGJ398	Pan-FGFR Reversible Type I	FGFR1: 0.9 FGFR2: 1.4 FGFR3: 1 FGFR4: 60	3TT0	Phase II NCT02706691	[50]
INCB054828	Pan-FGFR Reversible Type I	FGFR1: 0.4 FGFR2: 0.5 FGFR3: 1.2 FGFR4: 30	n/a	Phase II NCT03011372	[51]
Rogaratinib	Pan-FGFR Reversible Type I	FGFR1: 12–15 FGFR2: <1 FGFR3: 19 FGFR4: 33	n/a	Phase II/III NCT03410693	[52]
PRN1371	Pan-FGFR Irreversible Type I	FGFR1: 0.6 FGFR2: 1.3 FGFR3: 4.1 FGFR4: 19.3	n/a	Phase I NCT02608125	[53]
TAS-120	Pan-FGFR Irreversible Type I	FGFR1: 3.9 FGFR2: 1.3 FGFR3: 1.6 FGFR4: 8.3	6M2Q	Phase I/II NCT02052778	[54]
BLU-554	FGFR4 selective Irreversible, Type I	FGFR1: 624 FGFR2: 1202 FGFR3: 2203 FGFR4: 5	n/a	Phase I NCT02508467	[55]
H3B-6527	FGFR4 selective Irreversible, Type I	FGFR1: 320 FGFR2: 1290 FGFR3: 1060 FGFR4: <1.2	5VND	Phase I NCT02834780	[56]
FGF401	FGFR4 selective Reversible Covalent, Type I	FGFR1-3: >10,000 FGFR4: 1.1	6JPJ	Phase I/II NCT02325739	[13,57]

n/a stands for not available.
